# Plasma metabolic signatures of healthy dietary patterns and risk of metabolic dysfunction-associated steatotic liver disease and cirrhosis

**DOI:** 10.1007/s12072-026-11121-4

**Published:** 2026-07-22

**Authors:** Shijia Wang, Yingxin Liao, Zhijun Xue, Yuxiang Huang, Chao Yu, Xuechen Chen, Qiaoqiao Yang, Peiting Zhang, Mengyu Zhou, Yuqing Deng, Wenhua Ling, Xu Chen, Hongliang Xue

**Affiliations:** 1 The Key Laboratory of Advanced Interdisciplinary Studies, The First Affiliated Hospital of Guangzhou Medical University, No.1, Xinzao Road, Panyu District, Guangzhou, 511436 China; 2 Department of Nutrition, School of Public Health, Guangzhou Medical University, Guangzhou, China; 3 Guangzhou Institute of Respiratory Health, First Affiliated Hospital of Guangzhou Medical University, Guangzhou, China; 4 School of Nursing, Guangzhou Medical University, Guangzhou, China; 5 The Second School of Clinical Medicine, Guangzhou Medical University, Guangzhou, China; 6 Medical Examination Center, Third Affiliated Hospital, Sun Yat-Sen University, Guangzhou, China; 7 College of Pharmacy, Jinan University, Guangzhou, China; 8 Food Safety and Health Research Center, School of Public Health, Southern Medical University, Guangzhou, China; 9 State Key Laboratory of Ophthalmology, Zhongshan Ophthalmic Centre, Sun Yat-Sen University, Guangzhou, China; 10 School of Public Health/Guangdong Provincial Key Laboratory of Food, Nutrition and Health, Sun Yat-Sen University, No.74, Zhongshan Road 2, Yuexiu District, Guangzhou, 510080 China; 11 Department of Food Science and Nutrition, The Hong Kong Polytechnic University, No.11, Yucai Road, Hung Hom, Kowloon, Hong Kong SAR, China

**Keywords:** Healthy dietary pattern, Metabolomics, Metabolic signature, Metabolic dysfunction-associated steatotic liver disease, Cirrhosis, Dietary index for gut microbiota, Mediterranean-dietary approaches to stop hypertension diet intervention for neurodegenerative delay, Healthy plant-based diet index, Mediation analysis, Mendelian randomization, UK Biobank

## Abstract

**Background:**

The underlying mechanisms of the associations between dietary patterns and liver disease remain unclear. We aimed to identify metabolic signatures (MSs) reflecting adherence to ten healthy dietary patterns and to investigate their associations with metabolic dysfunction-associated steatotic liver disease (MASLD) and cirrhosis.

**Methods:**

This cohort study included 82,259 participants with detailed dietary and metabolomic data. MSs for each dietary pattern were derived using elastic-net regression. Cox proportional hazards regression, Mendelian randomization, and mediation analyses were employed to explore potential associations and mechanisms.

**Results:**

MSs for ten healthy dietary patterns were derived from 31 to 116 metabolites, primarily comprising fatty acids, lipids, and lipoprotein subclasses. Across all patterns, MSs were consistently associated with a lower risk of MASLD, with hazard ratios (HRs) ranging from 0.59 to 0.76. Notably, MSs for MIND, HPDI, rE-DII, and HLCD were associated with reduced cirrhosis risk (HRs: 0.56 to 0.63). Mendelian randomization analysis supported a potential causal relationship between MSs of MED, MIND, HPDI, and EAT-Lancet diets and liver diseases. Mediation analysis revealed that specific MSs accounted for 20.1% to 29.4% of the association between dietary patterns and MASLD, and 25.7% to 27.4% of that with cirrhosis. Metabolites from fatty acid metabolism and lipoprotein subclasses were significantly linked to liver diseases, and substantial mediated effects were observed across these metabolic pathways.

**Conclusions:**

Specific MSs linked to healthy dietary patterns are associated with reduced risk of liver disease, potentially underlying the diet's protective mechanism against MASLD and guiding future dietary guidelines in preventing progressive liver disease.

**Graphical Abstract:**

**Figure:**
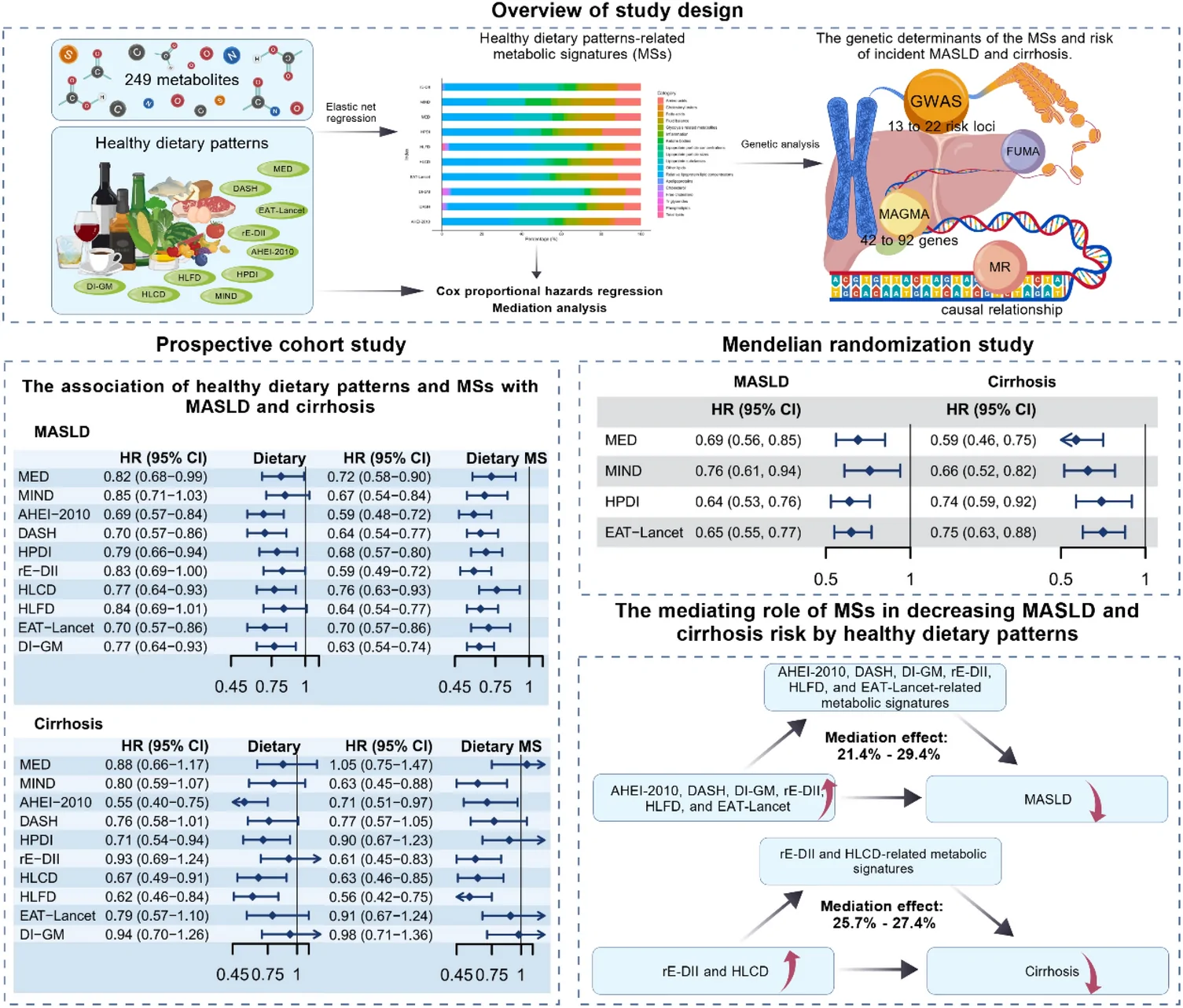

**Supplementary Information:**

The online version contains supplementary material available at 10.1007/s12072-026-11121-4.

## Introduction

Metabolic dysfunction-associated steatotic liver disease (MASLD) is the latest definition of non-alcoholic fatty liver disease (NAFLD). It is characterized by hepatic fat accumulation accompanied by at least one metabolic risk factor, such as obesity, type 2 diabetes, hypertension, or dyslipidemia [1]. MASLD is the most prevalent liver disease worldwide, currently affecting 38% of the global population [2]. Long-term progression of MASLD may lead to liver fibrosis, which can develop into cirrhosis and even liver cancer, ultimately resulting in death [3]. Globally, approximately 2 million people die from cirrhosis and liver cancer each year, accounting for 4% of total deaths and ranking as the 11th leading cause of mortality worldwide [4]. Given the absence of approved therapies specifically for MASLD, identifying modifiable risk factors is crucial for preventing the disease and mitigating its progression to severe complications.

The updated MASLD management guidelines released in 2024 emphasize that adopting a healthy diet is an essential non-pharmacological preventive measure for the onset and progression of MASLD [5]. Epidemiological evidence has demonstrated significant associations between various dietary patterns, such as the Mediterranean diet (MED), the EAT-Lancet diet, and the Dietary Approaches to Stop Hypertension (DASH), and the risk of MASLD and liver cirrhosis [6, 7]. However, methodological limitations are widely presented in traditional dietary questionnaire assessments, including recall bias and an inability to account for individual variations in nutrient absorption and metabolic capacity. In contrast, monitoring plasma metabolites can provide a more direct reflection of the impact of diet on the body's metabolic status [8]. Animal studies have shown that long-term high-sugar diets disrupt hepatic lipid metabolism in mice, accelerating the progression of MASLD [9]. In cases of overnutrition and obesity, alterations in hepatic fatty acid metabolism lead to triglyceride accumulation in hepatocytes, thereby promoting the development of MASLD [10]. Nonetheless, analyzing individual metabolites or single metabolic pathways alone cannot fully capture the complex effects of diet on liver health. To address these gaps, metabolomics-based nutritional research methods offer a more comprehensive approach to understanding the health effects of diet.

Metabolomics reflects the body's metabolic functional status by detecting a wide range of metabolites. In recent years, metabolomics approaches have been used to assess established dietary patterns, such as MED [11] and pro-inflammatory diets [12], and to investigate their relationships with adverse health outcomes. These studies provide robust support for a research framework based on the body's metabolic response. However, limited evidence exists regarding the association between healthy dietary patterns-related metabolic signatures (MS) and liver outcomes, and the impact of metabolic pathways in mediating the link between dietary adherence and liver health remains unclear. Therefore, our study employed the large UK Biobank cohort to identify MSs reflecting adherence to ten classic and new healthy dietary patterns and investigated their prospective associations with the risk of MASLD and cirrhosis. We then identified single-nucleotide polymorphisms (SNP) associated with the pattern-related MSs based on a genome-wide association study (GWAS) and applied Mendelian randomization (MR) to assess their causal relationship with liver outcomes. We further explored the mediating roles of these MSs and the inherent pathways. By combining metabolomics and genetics into nutritional research, this integrated approach aims to reveal the biological mechanisms linking healthy dietary patterns to the risk of MASLD and cirrhosis, explore predictive metabolic markers, and inform dietary recommendations for liver disease prevention.

## Materials and methods

### Study population

The UK Biobank is a large-scale, prospective cohort study of a significant population. Between 2006 and 2010, the study recruited approximately 500,000 participants aged 37 to 73 from England, Scotland, and Wales [13]. Eligible participants provided basic health-related information through touchscreen questionnaires, underwent physical measurements, and submitted biological samples. The UK Biobank was approved by the North West Multi-Center Research Ethics Committee (ref 21/NW/0157), and all participants provided written consent. We excluded individuals with missing dietary data, extreme total energy intake values, missing covariate data, pre-existing diseases at baseline, and missing metabolomics data, leaving 82,259 participants for the primary analyses (Fig. S1 and Table S1).

### Assessment of dietary pattern scores

Between April 2009 and June 2012, participants who provided email addresses were invited to complete the 24-h dietary recall questionnaire from the Oxford WebQ on five occasions [14]. The assessment covered 206 food items and 32 beverage items. The energy and macronutrient intake of each item was evaluated using the UK Nutrient Databank's food composition tables. For each participant, we calculated ten healthy dietary pattern scores, namely MED, mediterranean-dietary approaches to stop hypertension diet intervention for neurodegenerative delay (MIND), alternative healthy eating index 2010 (AHEI-2010), DASH, dietary index for gut microbiota (DI-GM), healthy plant-based diet index (HPDI), energy-adjusted diet inflammatory index (E-DII), healthy low-carbohydrate diet (HLCD), healthy low-fat diet (HLFD), and EAT-Lancet. The scoring criteria of dietary patterns are detailed in Supplementary Methods and Tables S2-S10. The E-DII score was reverse-coded (designated as rE-DII), with higher scores indicating lower inflammatory potential. All healthy dietary pattern scores were standardized to represent changes from the 10th to the 90th percentile.

### Assessment of metabolomics data

The nuclear magnetic resonance metabolomics data were obtained from Nightingale Health, which involved 249 metabolic biomarkers in EDTA plasma samples. Data were gathered from approximately 280,000 participants between June 2019 and April 2020 for the baseline assessment, and about 16,000 participants between April 2020 and June 2022 for the initial repeat assessment. Detailed information regarding the analytical procedures and quality control measures has been published online [15]. The metabolites encompass a wide range of metabolic pathways, including low-molecular-weight metabolites (such as amino acids, ketone bodies, and glycolytic metabolites), lipoprotein lipids (14 subclasses), as well as fatty acids and their composition.

### Assessment of outcomes

The study outcomes included MASLD and cirrhosis, defined using the International Classification of Diseases, Tenth Revision (ICD-10) or Operations/Procedure Codes, Version 4 (Table S11). The date and cause of death were obtained by linking to the death registries of the National Health Service (NHS) Information Centre (England and Wales) and the NHS Central Register (Scotland). Each participant contributed follow-up time from the date of registration to the date of hospital admission or death, or the end of follow-up (December 31, 2021, for England and Wales; February 28, 2022, for Scotland), whichever came first.

### Statistical analysis

#### Identification of MSs reflecting healthy dietary patterns

**Fig. 1:**
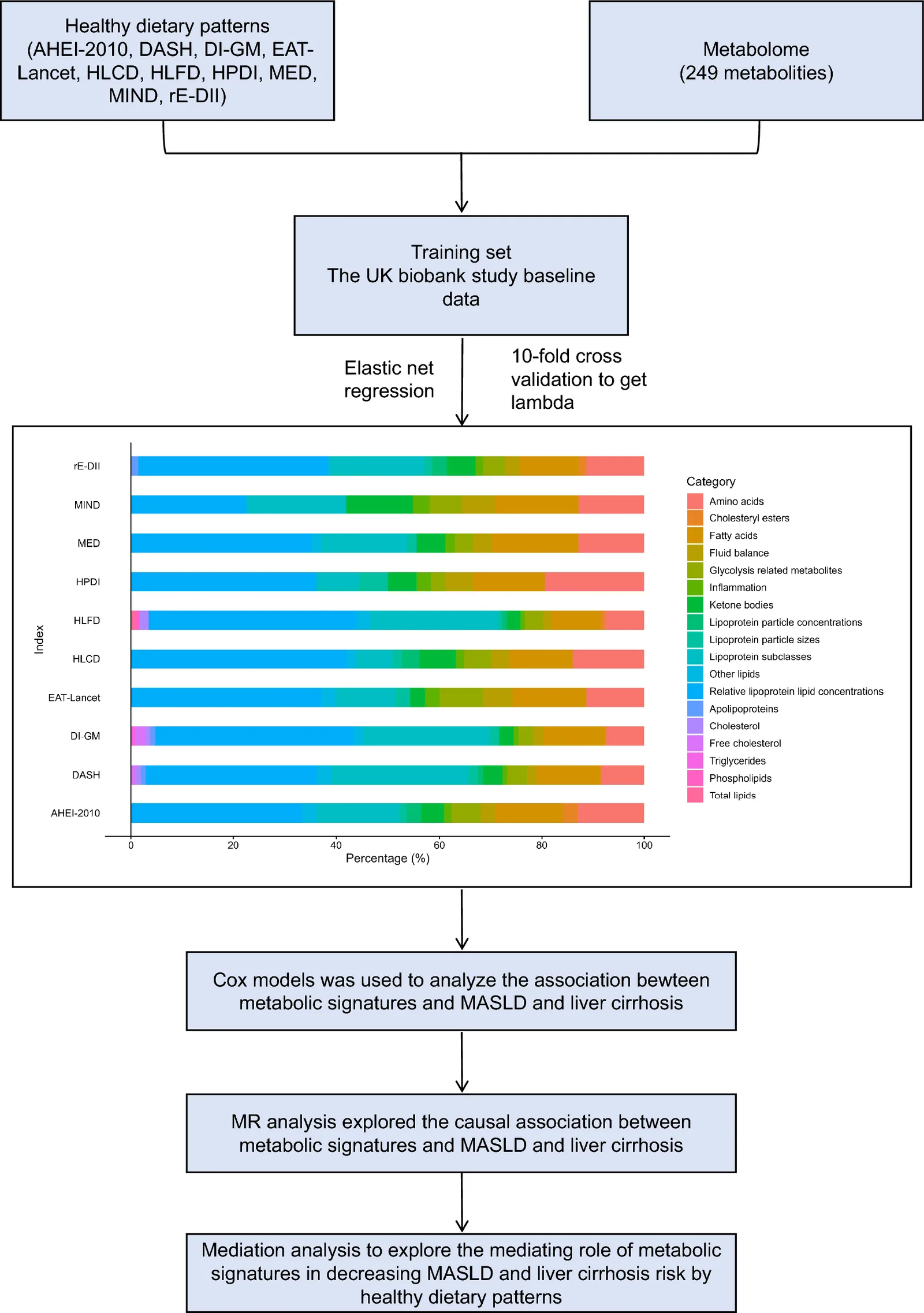
The metabolic signatures of healthy dietary patterns flow chart for analytic approach validation. *AHEI-2010* Alternative Healthy Eating Index 2010, *DASH* Dietary Approaches to Stop Hypertension, *DI-GM* Dietary Index for Gut Microbiota, *rE-DII* reverse Energy-adjusted Diet Inflammatory Index, *HLCD* Healthy Low-Carbohydrate Diet, *HLFD* Healthy Low-Fat Diet, *HPDI* Healthy Plant-Based Diet Index, *MED* Mediterranean Diet, *MIND* Mediterranean-Dietary Approaches to Stop Hypertension Diet Intervention for Neurodegenerative Delay

Using the UK Biobank's baseline metabolomics data as the training set, we identified metabolites reflecting adherence to healthy dietary patterns for liver outcomes research. Before conducting planned analyses, all 249 metabolites underwent log transformation and standardization to a common scale. Multivariable linear regression was employed to analyze the correlations between individual metabolites and each healthy dietary pattern score, with a Bonferroni-corrected *p* value < 0.05 considered statistically significant. Elastic net regression model (α = 0.5) with tenfold cross-validation, where the chosen lambda yielded a mean squared error within one standard error of the minimum, was then employed to construct the MSs of ten dietary patterns (Fig. 1). MSs were calculated by computing weighted sums of selected metabolites (coefficients ≠ 0), with weights corresponding to coefficients derived from elastic net regression (Supplementary Methods).

#### Associations of healthy dietary patterns and MSs with risk of MASLD and cirrhosis

We used multivariable-adjusted Cox proportional hazards regression models to calculate hazard ratios (HRs) and 95% confidence intervals (CIs) for the associations between healthy dietary pattern scores, their corresponding MSs, and the incidence of MASLD and cirrhosis fitted in three adjustment models (Supplementary Methods). The proportional hazards assumptions were verified using Schoenfeld residuals, and no significant violations were detected. Spearman's rank correlation coefficients were used to assess the correlations among MSs, healthy dietary pattern scores, and multiple food groups. Statistical significance was determined using Benjamini–Hochberg false discovery rate thresholds of 0.05. Covariate information (Table S12) and details of subgroup analysis and sensitivity analysis are described in the Supplementary Methods.

#### GWAS and MR analysis for MSs

A GWAS was conducted in 82,259 participants with both MS and genotype data. Variants with a minimum allele frequency of < 0.5% and minor allele frequency < 1% were removed. We performed a linear additive regression model in PLINK v2.0 to evaluate the association between MSs and SNPs, and applied the SNP2FUNC pipeline and MAGMA tool embedded in FUMA to perform functional mapping (Supplementary Methods). To investigate the potential causal effects of MSs on MASLD and cirrhosis, a two-sample MR analysis utilizing the inverse variance weighting (IVW) method was conducted (Supplementary Methods). A subsequent meta-analysis was then performed to combine the findings from the different studies, as detailed in the Supplementary Methods.

#### Mediation analysis for MSs and metabolic pathways

We conducted a mediation analysis to assess the mediated proportion for each MS and metabolic pathways constituting the overall signatures of the association between healthy dietary patterns and the development of MASLD and cirrhosis. The mediation analysis was adjusted for the covariates specified in Cox proportional hazards models.

All statistical analyses were performed using R software, version 4.5.1.

## Results

### Participant characteristics

**Fig. 2:**
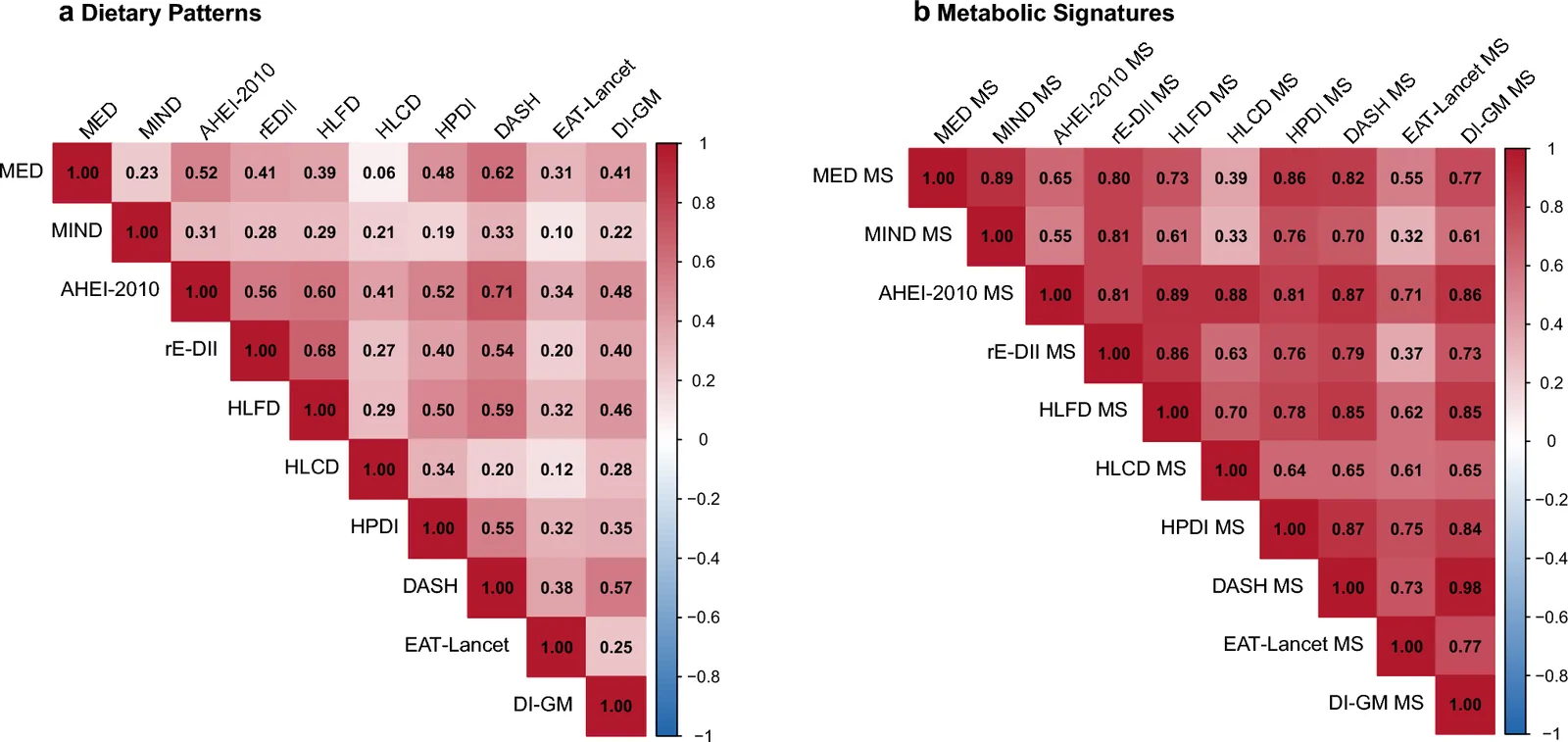
The partial spearman's rank correlations between different healthy dietary pattern scores or different dietary metabolic signatures. The partial Spearman's rank correlations between different energy-adjusted dietary patterns or different dietary metabolic signatures were estimated after adjustment for multivariable. *MS* Metabolic signature, *AHEI-2010* Alternative Healthy Eating Index 2010, *DASH* Dietary Approaches to Stop Hypertension, *DI-GM* Dietary Index for Gut Microbiota, *rE-DII* reverse Energy-adjusted Diet Inflammatory Index, *HLCD* Healthy Low-Carbohydrate Diet, *HLFD* Healthy Low-Fat Diet, *HPDI* Healthy Plant-Based Diet Index, *MED* Mediterranean Diet, *MIND* Mediterranean-Dietary Approaches to Stop Hypertension Diet Intervention for Neurodegenerative Delay

This study included 82,259 participants with metabolic data (Fig. S1). At baseline, the mean age was 55.97 ± 7.92 years, and 52.9% of the participants were female. During 10.2 person-years of follow-up per 100,000, we identified 775 cases of MASLD and 313 cases of cirrhosis. Compared to participants in the 10th percentile of dietary pattern scores, those in the 90th percentile were older and less likely to be current drinkers and smokers (Table S13). Participants in the highest percentile of dietary pattern scores generally had lower total energy intake, except for the MIND and DI-GM groups. Those who strongly adhered to AHEI-2010, DASH, HLFD, and HPDI were more likely to be female, while males tended to follow the other five dietary patterns, including the MED, MIND, HLCD, EAT-Lancet, and rE-DII. Except for the weak correlation between HLCD and MED (*r* = 0.06), the correlations among the healthy dietary pattern scores were generally moderate, ranging from 0.20 to 0.71 (Fig. 2A).

### Variation of metabolites in response to ten healthy dietary patterns

**Fig. 3:**
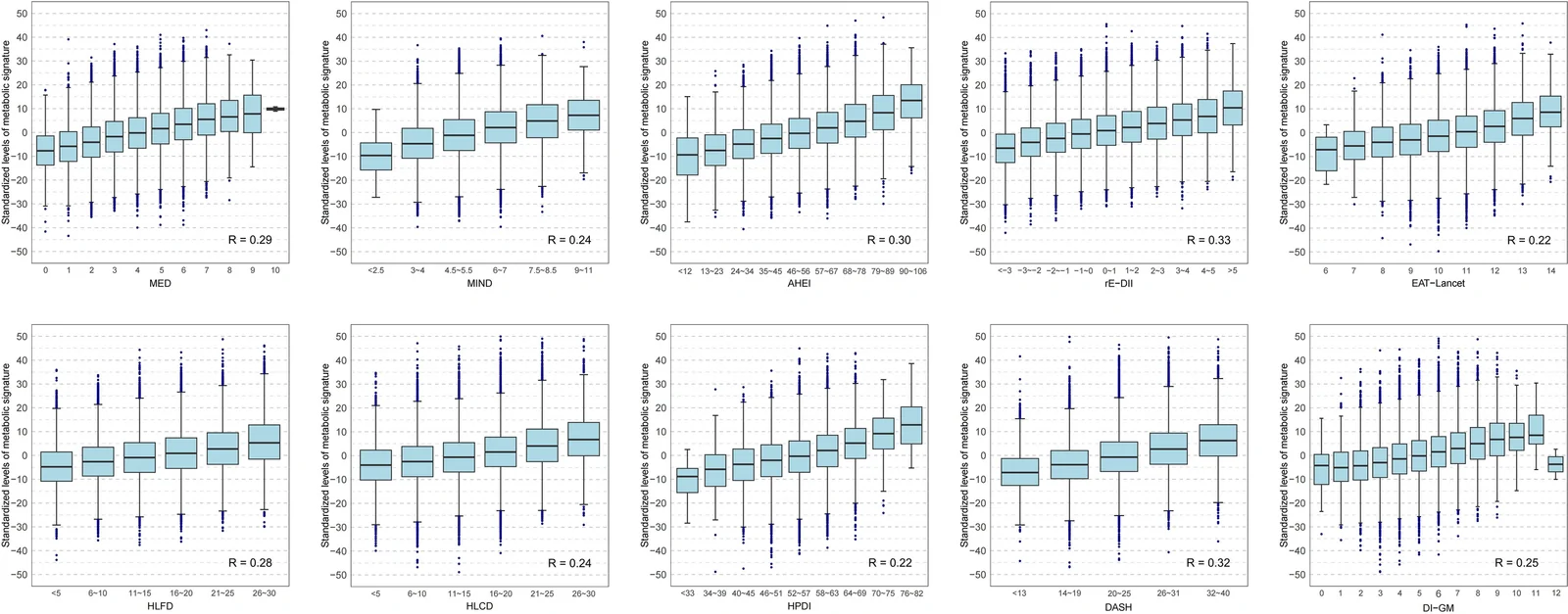
Correlations between each healthy dietary pattern and its metabolic signature (training set). The top and bottom edges of the box represented the third quartile and the first quartile of metabolic signature, respectively, and the horizontal line inside the box represented the median of metabolic signature. The whiskers extend from the box, representing the minimum and maximum values of the metabolic signature, with any points beyond this range considered outliers (i.e., blue-filled circles). *AHEI-2010* Alternative Healthy Eating Index 2010, *DASH* Dietary Approaches to Stop Hypertension, *DI-GM* Dietary Index for Gut Microbiota, *rE-DII* reverse Energy-adjusted Diet Inflammatory Index, *HLCD* Healthy Low-Carbohydrate Diet, *HLFD* Healthy Low-Fat Diet, *HPDI* Healthy Plant-Based Diet Index, *MED* Mediterranean Diet, *MIND* Mediterranean-Dietary Approaches to Stop Hypertension Diet Intervention for Neurodegenerative Delay

We analyzed 249 metabolites and found that most were significantly associated with healthy dietary pattern scores (Fig. S2), and the initial repeated measurements demonstrated high consistency (Fig. S3). Through elastic net regression for the baseline data, the MSs of the dietary pattern scores were derived from 31 to 116 metabolites. The pairwise correlation between different pattern-related MSs ranged from 0.32 to 0.98, with modest to strong correlations (Fig. 2B). Meanwhile, these MSs showed significant modest correlations with their corresponding healthy dietary pattern scores in both baseline (*r* = 0.22–0.30, all *p* < 0.001; Fig. 3) and repeated measure analyses (*r* = 0.19–0.35, all *p* < 0.001; Fig. S4). When further stratified by the status of incident health outcomes, the correlation coefficients between the MSs and the relevant dietary pattern scores remained broadly similar (Fig. S5A and B). Healthy dietary pattern scores and the corresponding MSs correlated similarly with food groups (Fig. S6 and 7).

**Fig. 4:**
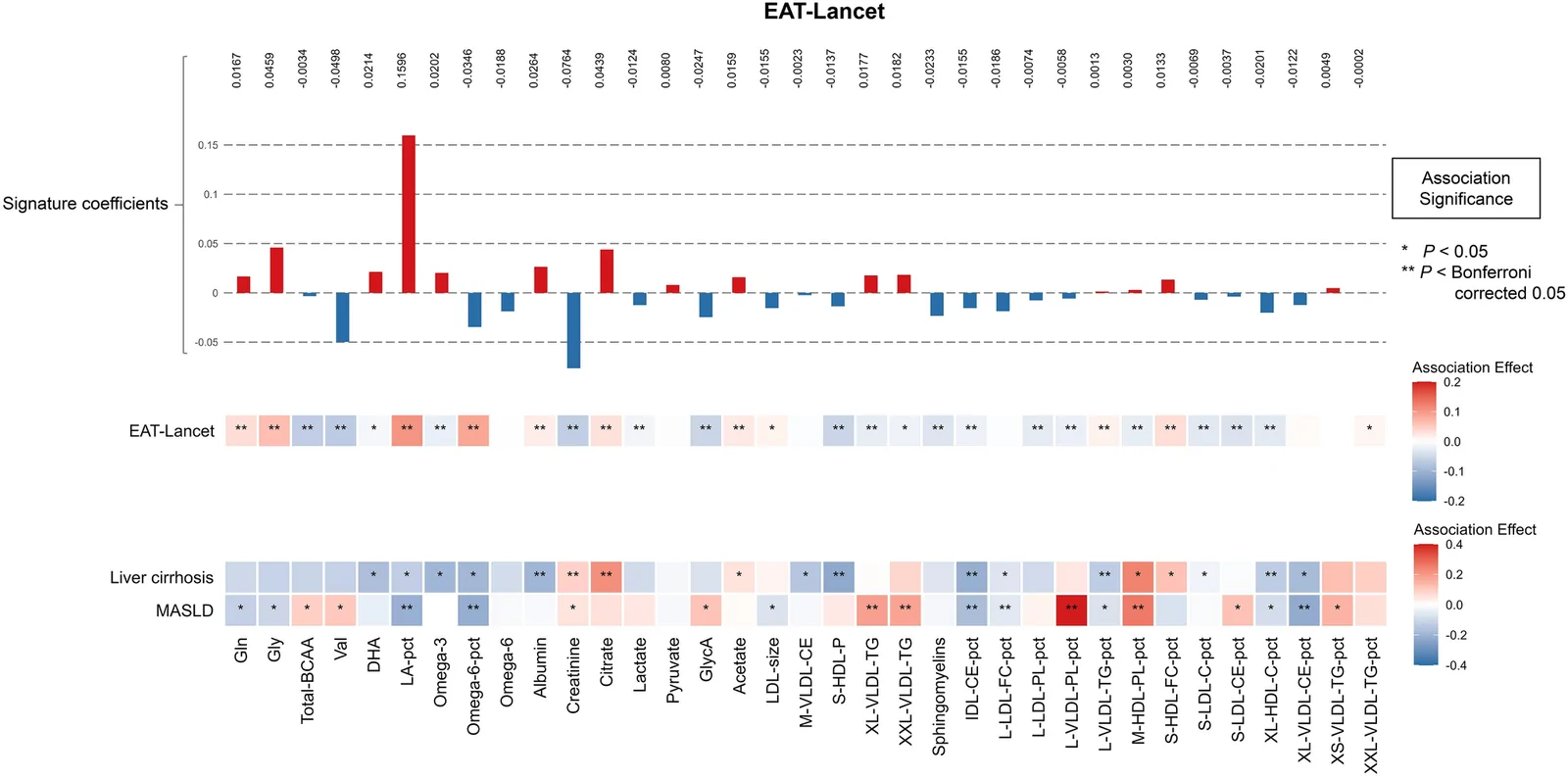
Associations between 35 metabolites constituting the metabolic signature and EAT-Lancet, MASLD, and cirrhosis. Presented from top to bottom are the coefficients (weights) of metabolites in the metabolic signature and associations with EAT-Lancet, MASLD, and cirrhosis. Coefficients for associations with healthy dietary patterns indicate the SD changes in metabolites per score increment of EAT-Lancet. Coefficients for the disease risk indicate the ln(hazard ratio) of disease risk per standard deviation increment in metabolites. *P < 0.05 and **Bonferroni-corrected *P* < 0.05. Colors depict the direction (blue-inverse and red-positive) and strength (the darker color, the stronger magnitude) of the associations. *MASLD* metabolic dysfunction-associated steatotic liver disease

Additionally, the selected metabolites exhibited extensive and consistent associations with ten healthy dietary pattern scores (Fig. 4, Fig. S8A–I, and Table S14). Among these, fatty acids demonstrated a significant contribution. For instance, omega-3 fatty acid was the main positive contributor to the MSs of MIND, DASH, DI-GM, and rE-DII, while docosahexaenoic acid (DHA) was the primary positive contributor to the MSs of MED, MIND, AHEI-2010, and HPDI. Linoleic acid (LA) showed the strongest positive contribution to the MSs of the AHEI-2010, DASH, and HLCD dietary patterns. Conversely, the percentage of saturated fatty acids (SFA%) exerted a significant negative influence on the MSs of AHEI-2010, rE-DII, HLCD, and HLFD. Notably, very low-density lipoprotein-triglyceride (VLDL-TG) and high-density lipoprotein-TG (HDL-TG) were negatively correlated with several dietary patterns.

### Associations between dietary patterns, MSs and risk of MASLD and cirrhosis

**Fig. 5:**
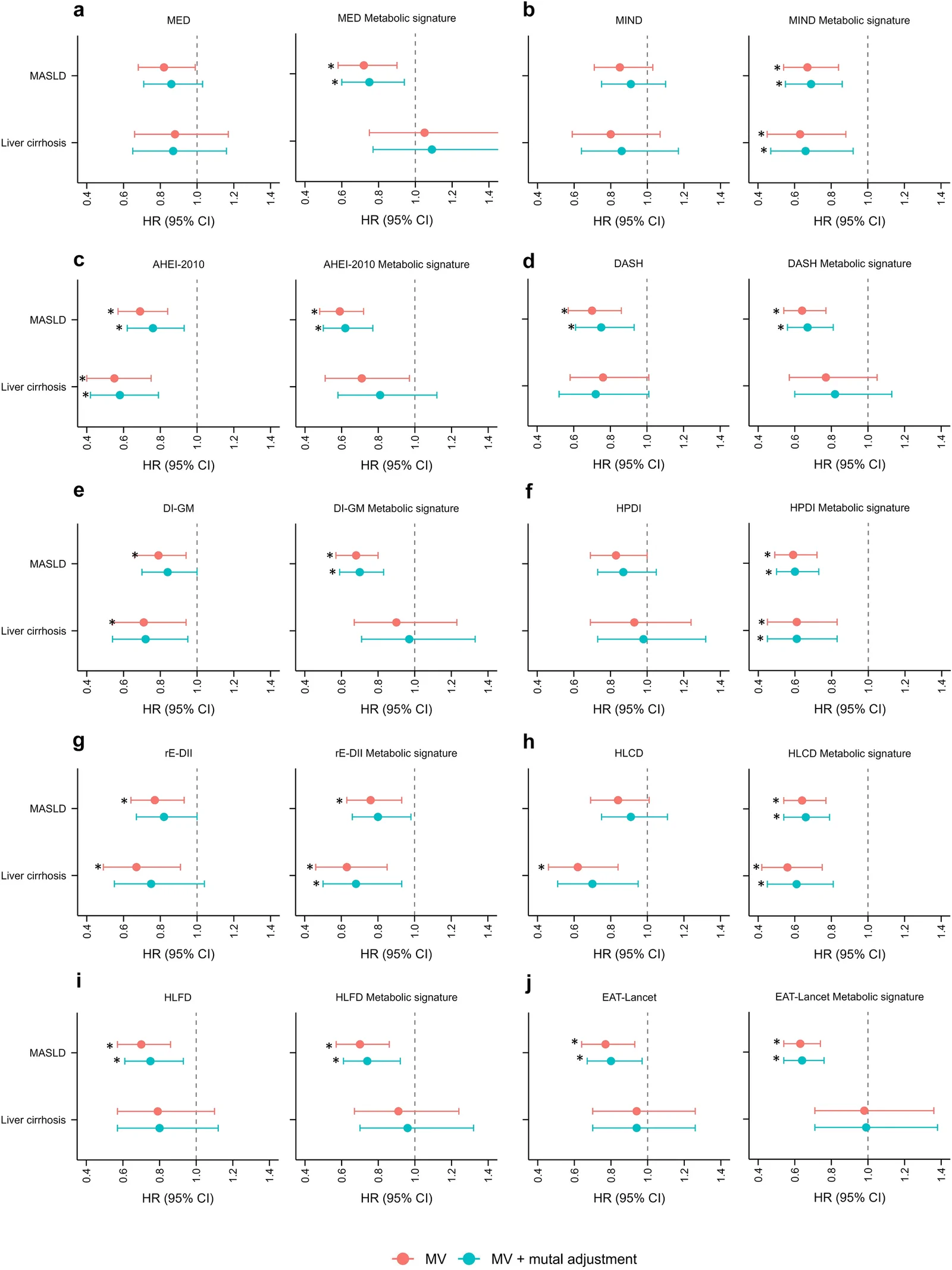
Associations of healthy dietary patterns and their corresponding metabolic signatures with risks of MASLD and cirrhosis. **a**–**j**, Dietary patterns and metabolic signatures for MED (**a**), MIND (**b**), AHEI-2010 (**c**), DASH (**d**), DI-GM (**e**), HPDI (**f**), rE-DII (**g**), HLCD (**h**), HLFD (**i**), and EAT-Lancet (**j**). The HRs comparing the 90th with the 10th percentile were represented by the squares, and the 95% CIs were represented by the error bars. The dashed lines represented HR = 1.0. The multivariable model was adjusted for age, sex, Townsend deprivation index, ethnicity, education, smoking status, physical activity, obesity, diabetes, hypertension, HDL cholesterol, and triglycerides. Total energy was adjusted for all dietary patterns and their metabolic signatures except rE-DII and its metabolic signature, and alcohol status was adjusted for DASH, DI-GM, HPDI, HLCD, HLFD, EAT-Lancet, and their metabolic signatures. For the mutual adjustment, each energy-adjusted dietary pattern and its corresponding metabolic signature were included simultaneously in the MV model to assess association independence. *The two-sided *P* values corrected using the FDR method were less than 0.05. *AHEI-2010* Alternative Healthy Eating Index 2010, *CI* confidence interval, *DASH* Dietary Approaches to Stop Hypertension, *DI-GM* Dietary Index for Gut Microbiota, *rE-DII* reverse Energy-adjusted Diet Inflammatory Index, *HLCD* Healthy Low-Carbohydrate Diet, *HLFD* Healthy Low-Fat Diet, *HPDI* Healthy Plant-Based Diet Index, *HR* hazard ratio, *MASLD* metabolic-associated steatotic liver disease, *MED* Mediterranean Diet, *MIND* Mediterranean-Dietary Approaches to Stop Hypertension Diet Intervention for Neurodegenerative Delay

When comparing the 90th with the 10th percentile of dietary pattern scores, AHEI-2010, DASH, DI-GM, rE-DII, HLFD, and EAT-Lancet were associated with a lower incidence of MASLD, with multivariable-adjusted HRs (95% CIs) of 0.69 (0.57–0.84), 0.70 (0.57–0.86), 0.79 (0.66–0.94), 0.77 (0.64–0.93), 0.70 (0.57–0.86), and 0.77 (0.64–0.93), respectively (Fig. 5 and Table S15). For cirrhosis, adherence to AHEI-2010, DI-GM, rE-DII and HLCD was associated with a 45%, 29%, 33% and 38% risk reduction, respectively. Based on the differences in HR values between pairs of scores and Z-tests, AHEI-2010 demonstrated a significantly superior advantage in preventing cirrhosis compared to MED, HPDI, and EAT-Lancet (Fig. S9A and C).

Meanwhile, the MSs of ten healthy dietary patterns were associated with a lower risk of MASLD in the comparison of the 90th versus the 10th percentile, with HRs (95% CI) of 0.72 (0.58–0.90), 0.67 (0.54–0.84), 0.59 (0.48–0.72), 0.64 (0.54–0.77), 0.68 (0.57–0.80), 0.59 (0.49–0.72), 0.76 (0.63–0.93), 0.64 (0.54–0.77), 0.70 (0.57–0.86), and 0.63 (0.54–0.74), respectively (Fig. 5 and Table S15). For cirrhosis, only the MSs of MIND, HPDI, rE-DII, and HLCD were inversely associated with a 37%, 39%, 37%, and 44% risk reduction, respectively. When both dietary pattern scores and the related MSs were included in the same models, the MSs of dietary patterns remained significantly inversely associated with the risk of MASLD, except for rE-DII (HR ranging from 0.60 to 0.75). On the other hand, the protective effects of MSs related to MIND, HPDI, rE-DII, and HLCD persisted on cirrhosis after adjustment for the respective dietary scores (HR ranging from 0.61 to 0.68). Additionally, HLCD-related MS showed a significantly superior advantage in preventing cirrhosis compared to the MSs of DI-GM, HLFD, and EAT-Lancet (Fig. S9B and D).

Sensitivity analyses and subgroup analyses showed similar results (Table S16–19). In the subgroup analyses, the inverse associations between diet-related MSs and MASLD were generally more pronounced in never-smokers, regularly physically active individuals, and those without obesity or diabetes. In addition, the associations between the MSs of AHEI-2010 and HLCD and the risk of cirrhosis were stronger in younger participants than in older individuals (Table S19). For individual food groups, higher consumption of sugary drinks was associated with an increased risk of MASLD. By contrast, increased consumption of tea and coffee was linked to a reduced risk of cirrhosis, whereas higher consumption of wine and spirits was associated with an increased risk of cirrhosis (Fig. S6). Major fatty acids that positively correlated with healthy dietary patterns were generally associated with reduced risk of liver disease, including omega-3 fatty acid, DHA and LA. Meanwhile, several lipoproteins that were negatively correlated with dietary patterns were adversely associated with liver outcomes, such as VLDL-TG and HDL-TG (Fig. 4 and Fig. S8A–I).

### Genetic correlates of MSs and risk of MASLD and cirrhosis

To explore the genetic basis of the identified MSs, we conducted a series of genetic analyses. GWAS revealed 13 to 22 risk loci and 19 to 75 lead SNPs associated with the pattern-related MSs (Fig. S10 and Table S20). MAGMA analysis identified 42 to 92 genes that were potentially involved in these signatures (Table S21), which were primarily attributed to lipid and lipoprotein metabolism, such as HDL function remodeling, cholesterol transport reversing, and triglyceride-rich lipoproteins clearance (Table S22). We also observed a tissue-specific enrichment in the liver (Fig. S11).

**Fig. 6:**
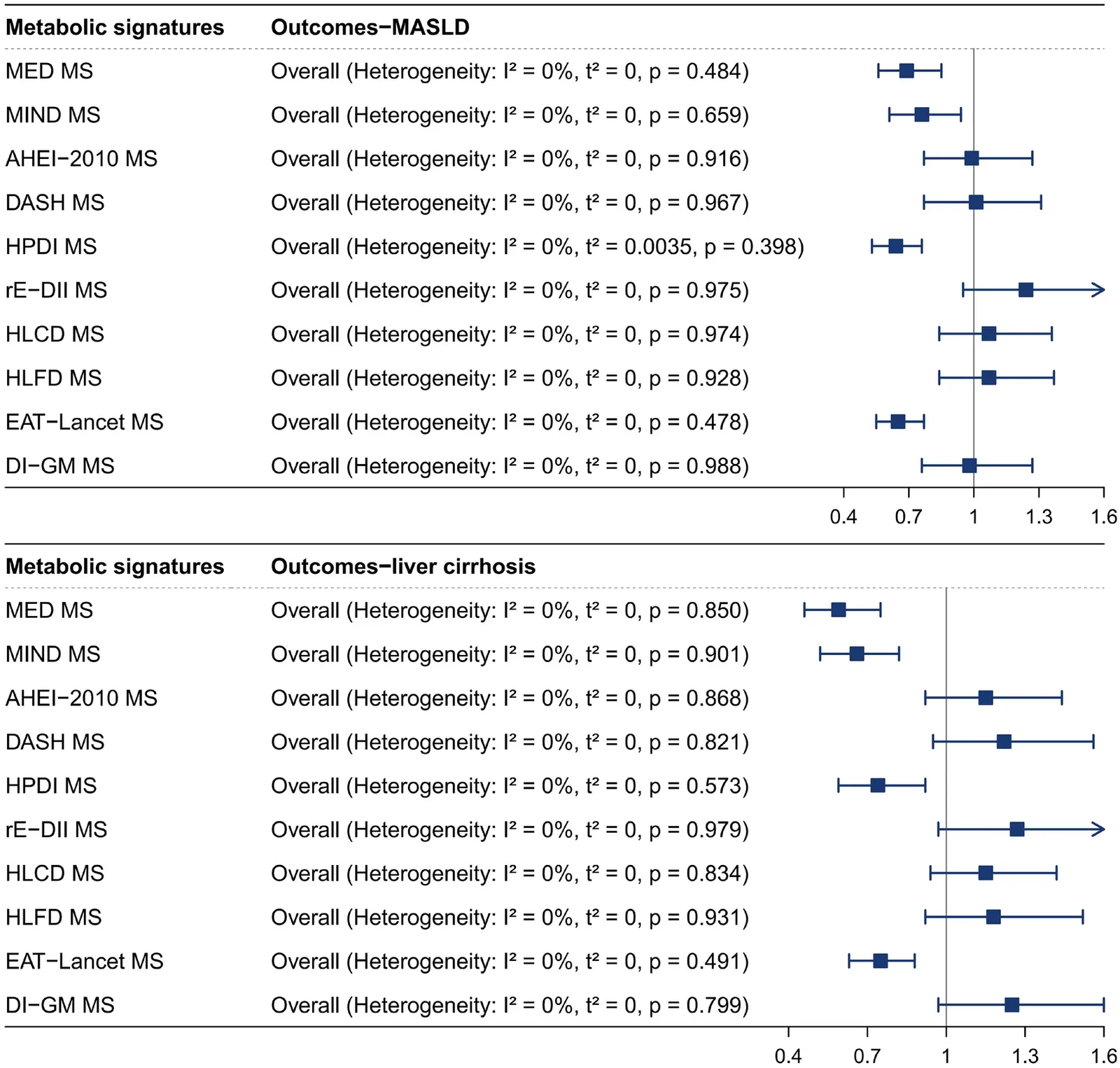
Mendelian randomization analysis of healthy dietary patterns-related metabolic signatures and MASLD and cirrhosis. Overall represents the comprehensive effect of meta-analysis on the MR analysis results of the different datasets (MASLD: finn-b-NAFLD, ebi-a-GCST90091033, ebi-a-GCST90054782; cirrhosis: ebi-a-GCST90018826, finn-b-CIRRHOSIS_BROAD). The dashed line represents the OR value of 1; the box point represents the OR value synthesized after meta-analysis. The horizontal line represents the size of the 95% CI. *P* value < 0.05 was considered statistically significant. *AHEI-2010* Alternative Healthy Eating Index 2010, *CI* confidence interval, *DASH* Dietary Approaches to Stop Hypertension, *DI-GM* Dietary Index for Gut Microbiota, *rE-DII* reverse Energy-adjusted Diet Inflammatory Index, *HLCD* Healthy Low-Carbohydrate Diet, *HLFD* Healthy Low-Fat Diet, *HPDI* Healthy Plant-Based Diet Index, *MASLD* metabolic-associated steatotic liver disease, *MED* Mediterranean Diet, *MIND* Mediterranean-Dietary Approaches to Stop Hypertension Diet Intervention for Neurodegenerative Delay, *OR* odds ratio

MR analysis further identified significant inverse associations between genetically instrumented MSs of MED, MIND, HPDI, and EAT-Lancet and susceptibility to both liver outcomes. The corresponding odds ratios (ORs) (95% CIs) were 0.69 (0.56, 0.85), 0.76 (0.61, 0.94), 0.64 (0.53, 0.76), and 0.65 (0.55, 0.77) for MASLD, and 0.59 (0.46, 0.75), 0.66 (0.52, 0.82), 0.74 (0.59, 0.92), and 0.75 (0.63, 0.88) for cirrhosis. (Fig. 6 and Table S23–24).

### Mediating role of MSs in decreasing MASLD and cirrhosis risk by dietary patterns

Mediation analysis was employed to assess the role of MSs in mediating the protective effects of healthy dietary patterns against MASLD and cirrhosis (Table S25-27). For MASLD, MSs significantly mediated the associations for AHEI-2010, DASH, DI-GM, rE-DII, HLFD, and EAT-Lancet diets, with the proportions of the total effect mediated being 29.4%, 23.7%, 26.7%, 23.1%, 21.2%, and 20.1%, respectively. These mediating effects were primarily attributed to metabolic pathways involved in fatty acid metabolism, lipoprotein subclasses, and inflammation (Fig. S12). In relation to cirrhosis, MSs related to rE-DII and HLCD were significant mediators, accounting for 27.4% and 25.7% of the associations, respectively. Fatty acid metabolism and lipoprotein subclasses were also the dominant pathways (Fig. S13).

## Discussion

In the present study, we identified and validated unique metabolite signatures reflecting adherence to 10 healthy dietary patterns derived from 31 to 116 metabolites. We found that all dietary pattern-related MSs were significantly associated with a decreased risk of MASLD. Meanwhile, the dietary pattern scores and MSs of MIND, HPDI, rE-DII, and HLCD were inversely associated with cirrhosis risk. Importantly, most protective associations between MSs and liver outcomes remained statistically significant even after adjusting for the relevant dietary pattern scores. MR analysis further supported a causal, protective role of the MSs related to MED, MIND, HPDI, and EAT-Lancet diets against both MASLD and cirrhosis. Through functional mapping, we found that the genetic correlates of MSs were significantly enriched in lipid and lipoprotein metabolism. Additionally, substantial mediating effects of MSs were observed between several dietary patterns and distinct liver outcomes, of which fatty acids, lipoprotein subclasses, and inflammation-related metabolites generally served as key mediators.

Previous studies have suggested that dietary patterns, as assessed by dietary questionnaires, may have a protective effect against liver disease [7, 16]. However, our study observed an inconsistent association between HPDI and MASLD risk, as reported by Ulzii BN et al. [17]. This discrepancy may be attributed to measurement error and reporting bias inherent in dietary recall questionnaires, which can influence the observed associations. In recent years, numerous studies have linked specific metabolites to the consumption of particular food groups, such as fish, meat, and legumes [18–20], suggesting that metabolites can effectively reflect dietary intake. Individuals' dietary habits are often complex, influenced by various food categories or adherence to dietary patterns, which lead to cumulative and distinct metabolic effects in the body. In this context, the construction of diet-related MSs provides a comprehensive approach to assessing an individual's overall adherence to various dietary patterns and their corresponding metabolic responses. Our study developed MSs for 10 traditional and emerging dietary patterns and explored their potential impact on liver health. The corresponding MSs comprised 31 to 116 metabolites, primarily derived from metabolic pathways related to fatty acids, amino acids, lipids, and lipoprotein subclasses. We observed correlations between the MSs and dietary patterns, as well as associations with specific food groups. The correlation between MSs and dietary pattern scores was moderate, consistent across repeated assessments, and independent of liver disease status. These findings suggest that diet-related MSs may serve as a reliable indicator of actual dietary intake. Furthermore, consistent with prior chronic disease research, the MS was inversely associated with liver disease risk, and this association was independent of the healthy dietary pattern score [11]. MR analysis provided additional evidence supporting the protective effect of MSs on liver health. Collectively, this study highlights the significant value of MSs in quantifying dietary exposure in nutritional research and the practical application in objectively predicting liver disease risk.

MSs significantly mediated the association between healthy dietary patterns and risk of liver disease. The overall MSs can reach a mediating proportion of approximately 20.1% to 29.4%, with fatty acid metabolism, inflammatory pathways, and lipoprotein subclasses contributing as key mediators. Notably, several representative fatty acids, such as omega-3 fatty acid, DHA, and LA, emerged as positive contributors to the construction of MSs. These fatty acids exhibited positive correlations with healthy dietary patterns and inverse associations with the risk of liver disease. Previously established interconnected biological mechanisms support these findings. N-3 polyunsaturated fatty acids (PUFA) can inhibit de novo lipogenesis, enhance mitochondrial fatty acid β-oxidation, and reduce hepatic inflammation [21, 22]. Furthermore, animal studies have indicated that increased intake of DHA [23] and LA [24] helped reduce hepatic steatosis. In contrast, SFA intake is associated with an increased risk of liver disease [25], with well-established evidence indicating that even-chain SFA group (including C14:0, C16:0, and C18:0) promotes liver inflammation [26]. Chronic inflammation can progress into pathological inflammation, disrupting tissue homeostasis and ultimately leading to fibrosis, cirrhosis, and even liver failure [27]. Additionally, certain lipoprotein characteristics, such as VLDL, VLDL-TG, and HDL-TG, were generally negatively correlated with healthy dietary intake and associated with an increased risk of MASLD and cirrhosis. Previous reports have indicated that elevated levels of VLDL-TG [28] and HDL-TG [29, 30] are associated with moderate to severe hepatic steatosis. Multiple healthy dietary patterns were correlated with lower circulating levels of VLDL-TG and HDL-TG, which may help reduce hepatic fat accumulation and slow disease progression. In summary, optimizing fatty acid composition, enhancing anti-inflammatory functions, and regulating lipoprotein metabolism may collectively contribute to improved liver health.

While the diet-related MSs shared common pathways, they also exhibited distinct effects. For instance, lipid and lipoprotein subclasses, fatty acids, and amino acids were major metabolic pathways involved in constructing the MSs. However, amino acids specifically mediated the associations between certain dietary patterns, including rE-DII, EAT-Lancet, DI-GM, and HLCD. The correlation between healthy dietary patterns and their corresponding MSs was moderate, but substantial variation in MSs was observed among participants with identical dietary scores. Moreover, the genetic loci associated with MSs were all involved in fatty acid metabolism (FADS1-3) [31], lipid and lipoprotein metabolism (APOE, APOA5, APOC1/C3, ABCA1) [32, 33], and inflammatory responses (MMP9, FNDC4) [34, 35] across 10 dietary patterns. The genetic underpinnings of metabolic disturbances were particularly enriched in lipoprotein metabolism pathways, with relevant genes being expressed in liver tissue. Although the number of identified lead SNPs for each MS varied widely, ranging from 19 to 75, the relevant gene function showed differences in more detailed mapping. These variations may be attributed to subtle differences in dietary composition, inter-individual metabolic variation, and genetically influenced differential responses to diet [36], contributing to the distinct effects on liver health. Plasma metabolomics can capture the overall metabolic homeostasis, reflecting the interaction of multiple factors that influence metabolism, thereby providing more precise biological insights than conventional dietary assessments. Based on these findings, the integration of metabolomic and genomic data not only provides clues for uncovering a multitude of potential liver-specific therapeutic targets but also establishes a scientific foundation for developing precision dietary interventions tailored to individual metabolic characteristics and genetic backgrounds, ultimately enabling the prevention of liver disease.

This study holds significant implications for clinical and public health practice. Firstly, by identifying 31 to 116 metabolites associated with healthy dietary patterns and establishing corresponding MSs, we laid the foundation for developing novel biomarkers and risk assessment tools that could be used for early screening and intervention of MASLD and cirrhosis. Secondly, we recommend increasing the intake of tea and coffee while reducing the consumption of wine, beer, spirits, and added sugars to reduce the risk of MASLD and cirrhosis. Thirdly, supplementation with omega-3/DHA-rich dietary products such as fish oil is suggested, which may help reduce hepatic lipid accumulation [37, 38]. Finally, synthesizing multiple analytical results, we particularly recommend the MIND and HPDI dietary patterns for the prevention of MASLD and cirrhosis. Overall, the integration of metabolomics and genomics provides a stronger evidence base for public health guidelines. This approach holds promise for shifting the focus of liver disease management from late-stage treatment to early, metabolically-informed risk intervention, thereby helping to curb its growing burden.

This study possesses several notable strengths, including a large sample size, a long-term follow-up period, extensive metabolomics data, the reliable selection of metabolites through elastic net regression, the application of MR analysis to explore causal relationships, and the combination of metabolomics and genetics in dietary research. However, our study also has some limitations. First, the Oxford WebQ collected dietary information based on self-reports, which may introduce recall bias. To mitigate the bias, we used the average values from multiple dietary assessments to provide a more accurate representation of overall dietary intake. Previous research has shown these questionnaires to be moderately to highly consistent between baseline and repeated visits [39]. While the main findings remained consistent among participants who completed more than one dietary assessment, changes in food consumption over the follow-up period could potentially weaken the observed findings. Second, demographic characteristics and lifestyle behaviors were collected at baseline; changes in these factors over time may attenuate the observed associations. Future studies with repeated measurements are needed to address this potential bias. Third, although metabolite measurements were comprehensive, they may still be subject to measurement errors or variability. The application of single-time-point metabolite measurements in the main analysis may not adequately capture long-term metabolic dynamics. However, we observed that the correlation between dietary pattern scores and MSs constructed using baseline metabolite data was similar to that using repeated metabolite measurements. Fourth, as no experimental investigations were conducted to identify molecular targets, the in-depth mechanistic interpretation of the MSs was limited. To uncover the underlying biological pathways, further well-designed experimental studies are needed to validate and clarify the relevant biological mechanisms and establish causal links between molecular targets and liver health. Finally, the study participants were of European descent. They had better health status than the general population, so it is necessary to validate the generalizability of the findings in more diverse populations.

## Conclusions

In summary, this large-scale prospective study systematically constructed and validated the metabolic signatures of ten healthy dietary patterns and further demonstrated the potential causal relationships associated with the MIND and HPDI-related MS with liver disease risk. These findings provide a scientific basis for developing metabolomics-based biomarkers of dietary adherence and, consequently, for formulating precise strategies for liver disease prevention.

## Supplementary Information

Below is the link to the electronic supplementary material.

Supplementary file1 (DOCX 17185 KB)

Supplementary file2 (XLSX 199 KB)

## Data Availability

The data that support the findings of this study are available from UK Biobank, but restrictions apply to the availability of these data, which were used under license for the current study, and so are not publicly available. The UK Biobank data can be accessed by researchers on the application (https://www.ukbiobank.ac.uk/).

## Abbreviations

AHEI-2010: Alternative Healthy Eating Index 2010
CI: Confidence interval
DHA: Docosahexaenoic acid
DASH: Dietary Approaches to Stop Hypertension
DI-GM: Dietary Index for Gut Microbiota
E-DII: Energy-adjusted Diet Inflammatory Index
FLI: Fatty liver index
GWAS: Genome-wide association study
HDL: High-density lipoprotein
HDS: Healthy diet score
HR: Hazard ratio
HPDI: Healthy Plant-Based Diet Index
HLCD: Healthy Low-Carbohydrate Diet
HLFD: Healthy Low-Fat Diet
IVW: Inverse variance weighting
LA: Linoleic acid
MED: Mediterranean Diet
MIND: Mediterranean-Dietary Approaches to Stop Hypertension Diet Intervention for Neurodegenerative Delay
MASLD: Metabolic Dysfunction-associated steatotic liver disease
MR: Mendelian randomization
MS: Metabolic signature
NAFLD: Non-alcoholic fatty liver disease
NHS: National Health Service
OR: Odds Ratio
PUFA: Polyunsaturated fatty acids
SD: Standard deviation
SFA%: Percentage of saturated fatty acids
SNP: Single-nucleotide polymorphism
TG: Triglyceride
VLDL: Very low-density lipoprotein
