# Supplementary material for: Plasma metabolic signatures of healthy dietary patterns and risk of metabolic dysfunction-associated steatotic liver disease and cirrhosis
Source: Hepatol Int. 2026 Jul 22;20(4):1058–72. doi: 10.1007/s12072-026-11121-4 (PMC13518410; doi:10.1007/s12072-026-11121-4)
Supplement: Supplementary file 1 [file 12072_2026_11121_MOESM1_ESM.docx]

**Plasma Metabolic signatures of Healthy Dietary Patterns and Risk of Metabolic Dysfunction-associated Steatotic Liver Disease and Other Chronic Liver Diseases: a Large Prospective Cohort Study from the UK Biobank**

**Shijia Wang1,2†, Yingxin Liao1,2†, Zhijun Xue1,3,4†, Yuxiang Huang5, Chao Yu6, Xuechen Chen7, Qiaoqiao Yang8, Peiting Zhang2, Mengyu Zhou2, Yuqing Deng9, Wenhua Ling10*, Xu Chen11*, Hongliang Xue1,2***

Contents

[Supplementary Method	3](#_Toc217303396)

[Fig. S1. Flow Chart of UK Biobank Participants Ultimately Included in This Study.	9](#_Toc217303397)

[Fig. S2. The volcano plot of the associations of 249 metabolites with healthy dietary patterns in the UK Biobank baseline data.	10](#_Toc217303398)

[Fig. S3. The volcano plot of the associations of 249 metabolites with healthy dietary patterns in the UK Biobank first repeated data.	12](#_Toc217303399)

[Fig. S4. Correlations between each healthy dietary pattern and its metabolic signature (testing set).	14](#_Toc217303400)

[Fig. S5. The partial Spearman’s rank correlations between healthy dietary patterns and corresponding dietary metabolic signatures stratified by the status of incident health outcomes (A) MASLD and (B) Liver cirrhosis.	16](#_Toc217303401)

[Fig. S6. The partial Spearman's rank correlations between healthy dietary patterns-related metabolic signatures and food groups in participants with metabolic data.	18](#_Toc217303402)

[Fig. S7. The partial Spearman’s rank correlations between the healthy dietary patterns and food groups.	20](#_Toc217303403)

[Fig. S8. Associations between 31-116 metabolites constituting the metabolic signatures and healthy dietary patterns, MASLD and liver cirrhosis.	29](#_Toc217303404)

[Fig. S9. Associations of ten healthy dietary patterns and Metabolic signatures with risk of incident MASLD and liver cirrhosis.	31](#_Toc217303405)

[Fig. S10. GWAS analysis of healthy dietary patterns related metabolites.	32](#_Toc217303406)

[Fig. S11. MAGMA Tissue Expression Analysis for metabolic signature.	35](#_Toc217303407)

[Fig. S12. Mediating effect by classification of metabolites in the association of healthy dietary patterns with MASLD risk.	36](#_Toc217303408)

[Fig. S13. Mediating effect by classification of metabolites in the association of healthy dietary patterns with liver cirrhosis risk.	38](#_Toc217303409)

[Reference	40](#_Toc217303410)

***Supplementary Method***

***Assessment of dietary patterns score***

The MED comprises 13 dietary components, with each element assigned a score ranging from 0 to 1 point depending on whether its consumption exceeds the validated UK dietary targets. The MIND, primarily developed to protect against cognitive impairment, evaluates 15 food components, awarding 0, 0.5, or 1 point per component based on adherence to recommended intake levels. The AHEI-2010 includes 11 dietary components, each scored from 0 to 10 points according to consumption level. The DASH allocates 0-5 points to each of the eight food components based on its consumption quintile. The DI-GM consists of 14 food items (the UK Biobank lacks data on chickpeas and cranberries), categorized into those beneficial to the gut microbiota and those unfavorable to it. Similarly, the HPDI allocates 0-5 points to each of the 17 food components based on its consumption quintile. To assess the long-term anti-inflammatory potential of diet, the intakes of 32 foods/nutrients available in the UK Biobank were used to compute the DII according to the scoring algorithm proposed by Shivappa et al [1]. The HLCD contains three components (percentage of energy from low-quality carbohydrate, unsaturated fat, and vegetable protein). HLFD contains three components (percentage of energy from high-quality carbohydrate, saturated fat, and vegetable protein). The HLCD and HLFD assign 0-11 points to each of the three components based on its sex-specific 11 quantiles. The EAT-Lancet score consists of 8 major dietary categories. Participants receive one point for meeting the recommendation of each category. Each dietary component is scored as either 0 or 1, with a total score ranging from 0 to 14 points.

***Identification of metabolic signature reflecting healthy dietary patterns***

Elastic net analysis was employed to analyze the associations between 249 metabolites and ten healthy dietary patterns. In the preliminary phase, elastic net regression models were applied to screen metabolites and construct distinct metabolic signatures for each of the healthy dietary patterns. This preferred approach combines the advantages of both lasso and ridge methods, effectively addressing multicollinearity, reducing overfitting, and selecting relevant features (2). We performed separate regression analyses for each healthy dietary pattern score with 249 named and standardized metabolites. The optimal lambda parameter was selected through 10-fold cross-validation, where the chosen lambda yielded a mean squared error within one standard error of the minimum. Furthermore, we calculated comprehensive metabolic signatures by computing weighted sums of selected metabolites (coefficients ≠0), with weights corresponding to coefficients derived from elastic net regression. These composite metabolic signatures represent the integrated effects of weighted sums of selected metabolites.

***Associations between the healthy dietary patterns, metabolic signature, and MASLD and liver cirrhosis risk***

The effects of healthy dietary patterns and metabolic signature on MASLD and liver cirrhosis were explored through three models: a) a crude model adjusted for age and sex (Model 1); b) a fully adjusted model that adjusted for self-reported ethnicity, Townsend deprivation index, education, physical activity, smoking status, obesity, diabetes, hypertension, high triglycerides, and low HDL cholesterol. All regression models for healthy dietary pattern scores and proteomic signatures were adjusted for total energy intake, except for the rE-DII model. For healthy dietary patterns without alcohol components (DASH, DI-GM, HPDI, HLCD, HLFD, and EAT-Lancet) and their corresponding metabolic signatures, additional adjustment was made for alcohol status (Model 2); and c) a multivariable model that incorporated both healthy dietary patterns and metabolic signatures for adjustment was used to explore the independent role of healthy dietary patterns and metabolic signatures on MASLD and liver cirrhosis (Model 3).

***Covariates***

Covariates were included age, sex (male, female), self-reported ethnicity (White/non-White), Townsend deprivation index (<-2.0, ≥-2.0 and <2.0, ≥2.0), education (less than high school, high school or equivalent, college or above), smoking status (never/previous/current), alcohol status (never/previous/current), obesity (none/yes), diabetes (none/yes), hypertension (none/yes), high triglycerides (none/yes), low HDL cholesterol (none/yes), and total energy intake. Regular physical activity was defined as at least 150 minutes of moderate activity per week, or at least 75 minutes of vigorous activity per week, or an equivalent combination (Table S12).

***Subgroup analysis***

To explore potential effect modifiers of healthy dietary pattern-related metabolic signatures on MASLD and liver cirrhosis, subgroup analyses were performed by age (<60 or ≥ 60 years), sex (male or female), smoking status (never, previous or current), physical activity (non-regular or regular), obesity (none or yes), and diabetes (none or yes). The likelihood ratio test evaluated interactions between stratification factors and healthy dietary pattern-related metabolic signatures on outcomes.

***GWAS and Mendelian randomization (MR) analysis for the metabolic signature***

The multiple regression function in PLINK2 was used for association analyses, adjusting for sex, age, and healthy dietary patterns (MED, MIND, AHEI-2010, DASH, DI-GM, HPDI, rE-DII, HLCD, HLFD, and EAT-Lancet), as well as the first 10 principal components of ancestry and the genotyping array. *p* < 5 × 10^ (-8) is considered statistically significant.

FUMA (https://fuma.ctglab.nl/, version 1.5.4) was used to perform function annotation for the GWAS results. Subsequently, MAGMA analysis was used to analyze individual genes and gene sets, and Bonferroni correction was applied to all tested genes. In this gene analysis, p < 2.63E-6 was considered to meet the genomewide significance. Tissue-specific analysis was performed using the RNAseq data set derived from GTEx (3).

MR analysis was employed to explore the relationship between metabolic signature and the risk of MASLD and liver cirrhosis. The published GWAS summary statistics datasets on MASLD and individual metabolites that constituted the metabolic signature were obtained from the MRC IEU OpenGWAS data infrastructure and the FinnGen biobank (Kurki MI, Karjalainen J, Palta P, et al.). FinnGen provides genetic insights from a well-phenotyped isolated population (4). The case numbers for MASLD were 8,434, 4,761, and 2,275, respectively, in the study by Ghodsian N (5), Fairfield Cj (6), and FinnGen biobank. In the Japan Biobank, 2,184 cases of liver cirrhosis were identified (7). SNPs related to metabolic signature with p value < 5×10-8 were selected as the instrumental variable, and LD clumping (r2 > 0.1) was used to eliminate the error caused by the chain imbalance to ensure the independence of the SNP. and “mr pleiotropy test” was used to analyze the pleiotropy of MR results; when p < 0.05, other factors were considered to play a role in the association between metabolic signature and MASLD and liver cirrhosis. A meta-analysis was used to synthesize the results of the three studies mentioned. The degree of heterogeneity was assessed based on the magnitude of I². I² < 50% was considered low heterogeneity. It was contemplated that the influence of heterogeneity between multiple studies on the overall effect was acceptable. The fixed effects model should be selected. On the contrary, I² > 50% indicated high heterogeneity qualitatively, and the random-effects model was therefore selected.

***Sensitivity analysis***

We conducted a series of sensitivity analyses to evaluate the robustness of our findings. First, we excluded patients who had completed the dietary questionnaire only once and analyzed only those who had completed it twice or more. Second, to minimize potential reverse causation, we re-analyzed the data after excluding participants who developed outcomes within the first 2 years of follow-up. Finally, we assessed the associations between dietary patterns and the risks of MASLD and other liver-related adverse events in 166,496 participants with complete dietary data.

**Fig. S1. Flow Chart of UK Biobank Participants Ultimately Included in This Study.**

Abbreviations: MASLD, metabolic-associated steatotic liver disease.

**Fig. S2. The volcano plot of the associations of 249 metabolites with healthy dietary patterns in the UK Biobank baseline data.**

**Fig. S3.** **The volcano plot of the associations of 249 metabolites with healthy dietary patterns in the UK Biobank first repeated data.**

**Fig. S4. Correlations between each healthy dietary pattern and its metabolic signature (testing set).** The top and bottom edges of the box represented the third quartile and the first quartile of metabolic signature, respectively, and the horizontal line inside the box represented the median of metabolic signature. The whiskers extend from the box, representing the minimum and maximum values of metabolic signature, with any points beyond this range considered as outliers (i.e., blue filled circles).

**Fig. S5. The partial Spearman’s rank correlations between healthy dietary patterns and corresponding dietary metabolic signatures stratified by the status of incident health outcomes (A) MASLD and (B) Liver cirrhosis.** The r in the figure represented the partial Spearman’s rank correlations between energy-adjusted dietary patterns and corresponding metabolic signatures, adjusting for age, sex, Townsend deprivation index, ethnicity, education, smoking status, physical activity, obesity, diabetes, hypertension, HDL cholesterol, and triglycerides. Total energy was adjusted for in the correlations between dietary patterns and corresponding metabolic signatures, except for rE-DII and its metabolic signatures, and alcohol status was adjusted for in the correlations between DASH, DI-GM, HPDI, HLCD, HLFD, EAT-Lancet, and their corresponding metabolic signatures. MASLD: metabolic dysfunction-associated steatotic liver disease.

**Fig. S6. The partial Spearman's rank correlations between healthy dietary patterns-related metabolic signatures and food groups in participants with metabolic data.** The partial Spearman's rank correlation coefficients between energy-adjusted dietary patterns and food groups after adjustment for multivariable factors are shown on the right side of the figure. The associations of food groups (comparing the 90th percentile with the 10th percentile) with MASLD and cirrhosis, adjusted for multivariable factors, are presented on the left side of the figure. The *P* values are reported, and only significant associations with two-sided *P* < 0.05 are highlighted in color, according to the magnitude of the HRs. *** *P* <0.001, ** *P* <0.01, * *P* <0.05. Abbreviation: HR, Hazard ratio; MASLD: metabolic dysfunction-associated steatotic liver disease.

**Fig. S7. The partial Spearman’s rank correlations between the healthy dietary patterns and food groups.** The partial Spearman’s rank correlations between different energy-adjusted dietary patterns were estimated after adjustment for age, sex, Townsend deprivation index, ethnicity, education, smoking status, physical activity, obesity, diabetes, hypertension, HDL cholesterol, triglycerides, total energy (except rE-DII proteomic signature), and alcohol status (including the DASH, DI-GM, HPDI, HLCD, HLFD, EAT-Lancet).

**Fig. S8. Associations between 31-116 metabolites constituting the metabolic signatures and healthy dietary patterns, MASLD and liver cirrhosis.** Presented from top to bottom are the coefficients (weights) of metabolites in the metabolic signature and associations with healthy dietary patterns, and MASLD and liver cirrhosis. Coefficients for associations with healthy dietary patterns indicate the SD changes in metabolites per score increment of healthy dietary patterns. Coefficients for the disease risk indicate ln(hazard ratio) of disease risk per standard deviation (SD) increment in metabolites. Asterisks denote the significance level of associations (**P* < 0.05 and **Bonferroni-corrected *P* < 0.05). Colors depict the direction (blue-inverse and red-positive) and strength (the darker color, the stronger magnitude) of the associations. MASLD: metabolic dysfunction-associated steatotic liver disease.

**Fig. S9. Associations of ten healthy dietary patterns and Metabolic signatures with risk of incident MASLD and liver cirrhosis.** The heatmaps show the HR difference between all pairs of scores. Positive differences are denoted in red and negative differences in green; a darker color indicates a greater difference. *Two-sided *P* < 0.05 based on a paired t-test comparing ORs (not adjusted for multiple comparisons).

**Fig. S10. GWAS analysis of healthy dietary patterns related metabolites.** (A) MED-related metabolic signature, (B) MIND-related metabolic signature, (C) AHEI-2010-related metabolic signature, (D) DASH-related metabolic signature, (E) DI-GM-related metabolic signature, (F) HPDI-related metabolic signature, (G) rE-DII-related metabolic signature, (H) HLCD-related metabolic signature, (I) HLFD-related metabolic signature, (J) EAT-Lancet-related metabolic signature. GWAS analysis of healthy dietary patterns related metabolites by Plink2, the multiple regression function was performed for association analyses by adjusting sex, age, healthy dietary patterns (MED, MIND, AHEI-2010, DASH, DI-GM, HPDI, rE-DII, HLCD, HLFD, EAT-Lancet), the first 10 principal components of ancestry, and genotyping array, *p* < 5 × 10^ (-8) was considered to be statistically significant. The red line represents a p-value threshold of 5 × 10^ (-8).

**Fig. S11. MAGMA Tissue Expression Analysis for metabolic signature.** (A) MED-related metabolic signature, (B) MIND-related metabolic signature, (C) AHEI-2010-related metabolic signature, (D) DASH-related metabolic signature, (E) DI-GM-related metabolic signature, (F) HPDI-related metabolic signature, (G) rE-DII-related metabolic signature, (H) HLCD-related metabolic signature, (I) HLFD-related metabolic signature, (J) EAT-Lancet-related metabolic signature. MAGMA analyzed Tissue Expression Analysis for metabolic signature. Tissue-specific analysis was performed using the RNAseq data set derived from GTEx. Bonferroni-corrected *p* < 0.05 was considered statistically significant. The dotted line indicates statistical significance, and Bonferroni-corrected *p* < 0.05 are highlighted in red.

**Fig. S12. Mediating effect by classification of metabolites in the association of healthy dietary patterns with MASLD risk.** Colors denote the degree of intermediation and magnitudes. *P-*value less than 0.05 was considered statistically significant. *** *P* <0.001, ** *P* <0.01, * *P* <0.05. Abbreviations: AHEI-2010, Alternative Healthy Eating Index 2010; DASH, Dietary Approaches to Stop Hypertension; DI-GM, Dietary Index for Gut Microbiota; rE-DII, reverse Energy-adjusted Diet Inflammatory Index; HPDI, Healthy Plant-Based Diet Index; HLCD, Healthy Low-Carbohydrate Diet; HLFD, Healthy Low-Fat Diet; MED, Mediterranean Diet; MIND, Hypertension Diet Intervention for Neurodegenerative Delay; MASLD, metabolic-associated steatotic liver disease.

**Fig. S13. Mediating effect by classification of metabolites in the association of healthy dietary patterns with liver cirrhosis risk.** The role of metabolite classification in the association between healthy dietary patterns and liver cirrhosis was analyzed using a mediation effect model that employs mediation analysis for survival data. Colors denote the degree of intermediation and magnitudes; asterisks represent association significance, *** *P* <0.001, ** *P* <0.01, * *P* <0.05. *P-*value less than 0.05 was considered statistically significant. MASLD, metabolic-associated steatotic liver disease.

**Reference**

[1] Shivappa N, Steck SE, Hurley TG, et al. Designing and developing a literature-derived, population-based dietary inflammatory index. Public Health Nutr. 2014;17:1689-1696.
[2] Zou H, Hastie T. Regularization and variable selection via the elastic net. J R Stat Soc: Ser B. 2005;67:301-320.
[3] Watanabe K, Taskesen E, van Bochoven A, Posthuma D. Functional mapping and annotation of genetic associations with FUMA. Nat Commun 2017;8:1826.
[4] Kurki MI, Karjalainen J, Palta P, et al. FinnGen provides genetic insights from a well phenotyped isolated population. Nature 2023;613:508-518.
[5] Ghodsian N, Abner E, Emdin CA, et al. Electronic health record-based genome-wide meta-analysis provides insights on the genetic architecture of non-alcoholic fatty liver disease. Cell Reports Medicine 2021;2.
[6] Fairfield CJ, Drake TM, Pius R, et al. Genome‐Wide Association Study of NAFLD Using Electronic Health Records. Hepatology Communications 2021;6:297 308.
[7] Sakaue S, Kanai M, Tanigawa Y, et al. A cross-population atlas of genetic associations for 220 human phenotypes. Nat Genet. 2021;53:1415-1424.
